# Effect of bone-marrow-derived mesenchymal stem cells on high-potential hepatocellular carcinoma in mouse models: an intervention study

**DOI:** 10.1186/2047-783X-18-34

**Published:** 2013-09-30

**Authors:** Tianran Li, Bin Song, Xiangke Du, Zhengmao Wei, Tianlong Huo

**Affiliations:** 1 Department of Radiology, The 95th Hospital of PLA, PuTian, FuJian Province 351100, China; 2 Department of Radiology, Peking University People’s Hospital, Beijing 100044, China

**Keywords:** Bone-marrow-derived mesenchymal stem cells, Osteopontin, Bone sialoprotein, Integrins, High metastatic potential cell, Animal model

## Abstract

**Background:**

There are two completely contradictory views regarding the impact of human bone-marrow-derived mesenchymal stem cells (hMSCs) on hepatocellular carcinomas (HCCs). The aim of this study was to investigate the effect of hMSC engraftment on HCC tissues in nude mouse models, and assess the effect on metastatic potential of HCC.

**Methods:**

hMSCs were engrafted into the nude mouse models of high metastatic HCC via the tail vein. The mice in the experimental group were engrafted with hMSCs (5 × 10^5^ cells per mouse) via the tail vein 15 days after inoculation of tumor cells, twice a week, while the animals in the control group were injected with hMSC culture medium (0.2 mL per mouse) via the tail vein. The subcutaneous tumor size was measured using an electronic digital caliper once every 4 days after hMSC engraftment. After 2, 3, 4, 5 and 6 weeks of tumor cell inoculation, the mice were killed and the tumors were collected in their entirety. The tumor weights and body weights of mice were measured, and the tumor inhibition rate was calculated. Quantitative real-time polymerase chain reaction (RT-PCR) was used to determine the expression of metastasis-related genes including osteopontin (OPN), bone sialoprotein (BSP) and integrin α5 subunit (α-V) in the mouse models of high-metastatic HCC, and the expression of apoptosis-related genes including B cell lymphoma/leukemia-2 (Bcl2), Bcl-2 associated X protein (Bax) and caspase 3 in tumor samples.

**Results:**

The tumor weight inhibition rate was 26.62% at 2 weeks, 52.00% at 3 weeks, 38.20% at 4 weeks, 31.98% at 5 weeks, and 30.23% at 6 weeks. Tumor tissue weight comparison results were significantly lower in the hMSC engraftment groups than in the control group at the second and third weeks. The expression of metastasis-related factors OPN, BSP and α-V gene was downregulated with time. The expression of antiapoptotic gene Bcl2 exhibited an obvious declining tendency, while the expression of apoptotic genes Bax and caspase 3 showed an obvious rising tendency. The expression of α-V and BSP significantly correlated positively with the expression of Bcl2, and negatively correlated with the expression of Bax and caspase 3. The tumor inhibition rate was not significantly correlated with the expression of antiapoptotic and apoptotic factors, and α-V and BSP factors, though it exhibited a significantly negative correlation with the expression of OPN.

**Conclusions:**

The highest tumor inhibition rate was observed 3 weeks after hMSCs engraftment, and the tumor inhibition rate gradually reduced with the progression of time. The metastatic potential of tumor cells was downregulated after hMSC engraftment and hMSCs induce further tumor cells apoptosis. The decrease in the proliferation ability of tumor cells may induce a decline in metastatic potential in tumor cells.

## Background

Hepatocellular carcinoma is one of the most common cancers, with poor prognosis and a high recurrence rate; metastatic recurrence is the major obstacle to improve the prognosis of HCC patients. Recently, the biological treatment of HCC has become a hot research area [1]. Human bone-marrow-derived mesenchymal stem cells (hMSCs) are a type of adult stem cell with multilineage differentiation potential. hMSCs are closely associated with multiple cells in liver tissues, and can differentiate into oval cells, fibroblasts and myofibroblasts in the liver, which exhibit functions of repairing injured liver tissues [2]. Autologous bone-marrow stem-cell transplantation has been used for treatment of decompensated hepatic cirrhosis, which proves that stem cell transplantation is a novel, safe and effective approach [3]. hMSCs exhibit chemotaxis and homing functions and present a high efficacy of promoting the reconstruction of the immune system, removing residual lesions and preventing recurrence and metastasis [4]. However, the opposite view also exists. Researchers believe that hMSCs can promote the growth and metastasis of hepatocellular carcinoma cells [5]. In order to investigate intervention effects of hMSCs with regard to hepatocellular carcinoma tissues, in the present study hMSCs were engrafted into animal models of hepatocellular carcinoma (HCC) with high-metastatic potential. The changes in metastasis potential were then observed.

## Methods

The work for this study was undertaken in the Gynecology Key Laboratory of Peking University People’s Hospital from May 2010 to May 2012.

### Cell lines

hMSCs were purchased from Cyagen Biosciences (Guangzhou) Inc. (Guangzhou, China). Human HCC cells with high metastatic potential (high-metastatic hepatocellular carcinoma cell 97 (MHCC97-H)) were supplied by the Liver Cancer Institute of Fudan University (Shanghai, China).

### Experimental animals

A total of 36 6-week-old specific-pathogen-free (SPF) grade nude mice of the Balb/c strain (female:male ratio of 1:1), each weighing 14 to 17 g, were purchased from the Laboratory Animal Center of the National Institutes for Food and Drug Control (Beijing, China). Animals were kept within the animal care facility of the Peking University Health Science Center. The experiments conform to the Guide for the Care and Use of Laboratory Animals published by the US National Institutes of Health (NIH Publication No. 85–23, revised 1996). The housing and care procedures in the study were performed in accordance with the guidelines and regulations composed by the Animal Care Committee of the University of Peking University Health Science Center and approved by the Institutional Animal Care and Use Committee of the Peking University Health Science Center, China.

### Establishment of nude mouse model of HCC with high metastatic potential

Normally cultured MHCC97-H cells were prepared into a single-cell suspension at a concentration of 2 × 10^5^ cells/mL using a routine technique. Approximately 0.8 mL of the cell suspension was sampled under aseptic conditions and was subcutaneously seeded onto the armpit of the upper extremities of nude mice.

### Grouping and intervention methods

**Figure 1:**
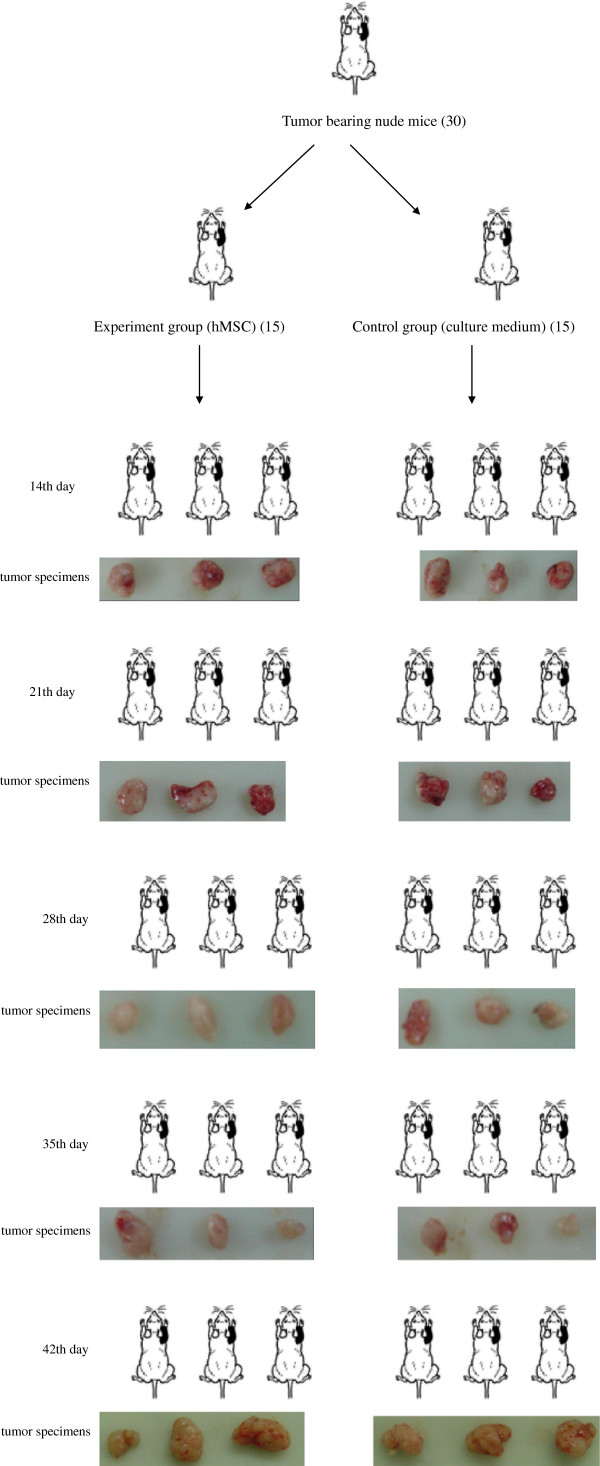
**Experiment design groups and works flow methods.** The schematic diagram shows a group situation. Photographs are tumor tissue specimens *in vitro* at different timepoints.

Mice were randomly assigned into one of two groups (n = 15 animals per group), and all mice were weighed and numbered. The mice in the experimental group were engrafted with hMSCs (5 × 10^5^ cells per mouse) via the tail vein 15 days after inoculation of tumor cells, twice a week for 6 weeks successively, while the animals in the control group were injected with hMSC culture medium (0.2 mL per mouse) via the tail vein at the same time. The subcutaneous tumor size was measured using an electronic digital caliper once every 4 days after hMSC engraftment. After 2, 3, 4, 5 and 6 weeks of tumor cell inoculation, the mice were killed and the tumors were collected in their entirety. The tumor weight and body weight of mice were measured (see Figure 1).

### Data processing method

The tumor inhibition rate was calculated using the following formula:

Tumorinhibitionrate%＝1‒EgCg×100%

E(g): mean tumor weight in the experiment group; C(g): mean tumor weight in the control group.

### Determination of expression of osteopontin (OPN), bone sialoprotein (BSP), integrin α5 subunit (α-V), B cell lymphoma/leukemia-2 (Bcl2), Bcl-2 associated X protein (Bax) and caspase 3 in tumor samples from animal models of high metastatic HCC using quantitative real-time polymerase chain reaction (RT-PCR)

The primers for RT-PCR amplification were designed according to the sequences in the GenBank database (Table 1), which were synthesized by the Cyagen Biosciences (Guangzhou) Inc. (Guangzhou, China). Human β-actin gene served as the internal control. Total RNA was isolated from tumor samples, and then reversely transcribed into cDNA using RevertAid™ M-MLV reverse transcriptase and Oligo(dT). The corresponding cDNA was amplified using a RT-PCR technique based on specific primers (Table 1), with predegeneration at 95°C for 5 minutes, followed by 30 cycles of degeneration at 94°C for 30 s, annealing at 58°C for 30 s, extension at 72°C for 30 s, and the final elongation at 72°C for 10 minutes. The PCR amplification product was electrophoresed, and semiquantitative analysis of the PCR amplification product was performed using a gel electrophoresis processing system.

**Table 1:** Sequences of primers for real-time polymerase chain reaction (RT-PCR) amplification

**Gene**	**Sequence of primer**
Osteopontin (OPN)	Forward primer: 5′-TCACCAGTCTGATGAGTCTCAC-3′
	Reverse primer: 5′-CAGGTCTGCGAAACTTCTTAGAT-3′
Bone sialoprotein (BSP)	Forward primer: 5′-GAATGGCCTGTGCTTTCTCAA-3′
	Reverse primer: 5′-TCGGATGAGTCACTACTGCCC-3′
Integrin α5 subunit (α-V)	Forward primer: 5′-CCTCAGACGCTGCGTGGAGC-3′
	Reverse primer: 5′-AGGGCTGAGCTTCGGAGCGA-3′
B cell lymphoma/leukemia-2 (Bcl2)	Sense primer: 5′-ATCCAGGATAACGGAGGC-3′
	Antisense primer: 5′-CAGCCAGGAGAAATCAAAC-3′
Bcl-2 associated X protein (Bax)	Sense primer: 5′-GACCCGGTGCCTCAGGATGC-3′
	Antisense primer: 5′-GTCTGTGTCCACGGCGGCAA-3′
Caspase 3	Sense primer: 5′-CATGGAAGCGAATCAATGGACT-3′
	Antisense primer: 5′-CTGTACCAGACCGAGATGTCA-3′
β-Actin	Forward primer: 5′-GCAGAAGGAGATCACAGCCCT-3′
	Reverse primer: 5′-GCTGATCCACATCTGCTGGAA-3′

### Statistical analysis

Tumor inhibition rates were calculated, and a graph of the tumor inhibition rates change over time was drawn. The tumor weights were compared between groups at different timepoints.

All statistical analyses were performed using the SPSS statistical software version 16.0 (SPSS Inc., Chicago, IL, USA). The Student *t* test was used to compare the difference between groups, with a *P* value <0.05 indicative of statistical significance.

## Results

### Effect of hMSCs on transplanted tumors in nude mice produced by inoculation of MHCC97-H cells

The tumor inhibition rate was calculated according to the tumor tissue specimen weights *in vitro* before and after hMSC intervention. The experimental results show that the tumor weight inhibition rate was 26.62% at 2 weeks, 52.00% at 3 weeks, 38.20% at 4 weeks, 31.98% at 5 weeks and 30.23% at 6 weeks.

**Figure 2:**
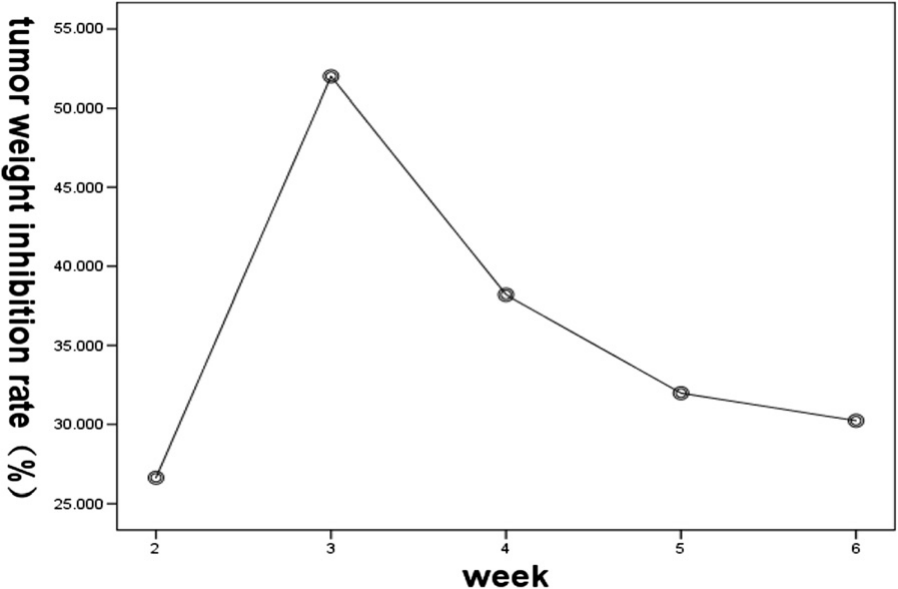
The changes of tumors weight inhibition rate following engraftment of human mesenchymal stem cells (hMSCs) over time.

The changes in transplanted tumors in nude mice produced by inoculation of MHCC97-H cells following engraftment of hMSCs for successive 6 weeks are shown in Figure 2. It was observed that the highest tumor inhibition rate was seen 3 weeks after hMSC engraftment, and the tumor inhibition rate gradually reduced with time.

**Figure 3:**
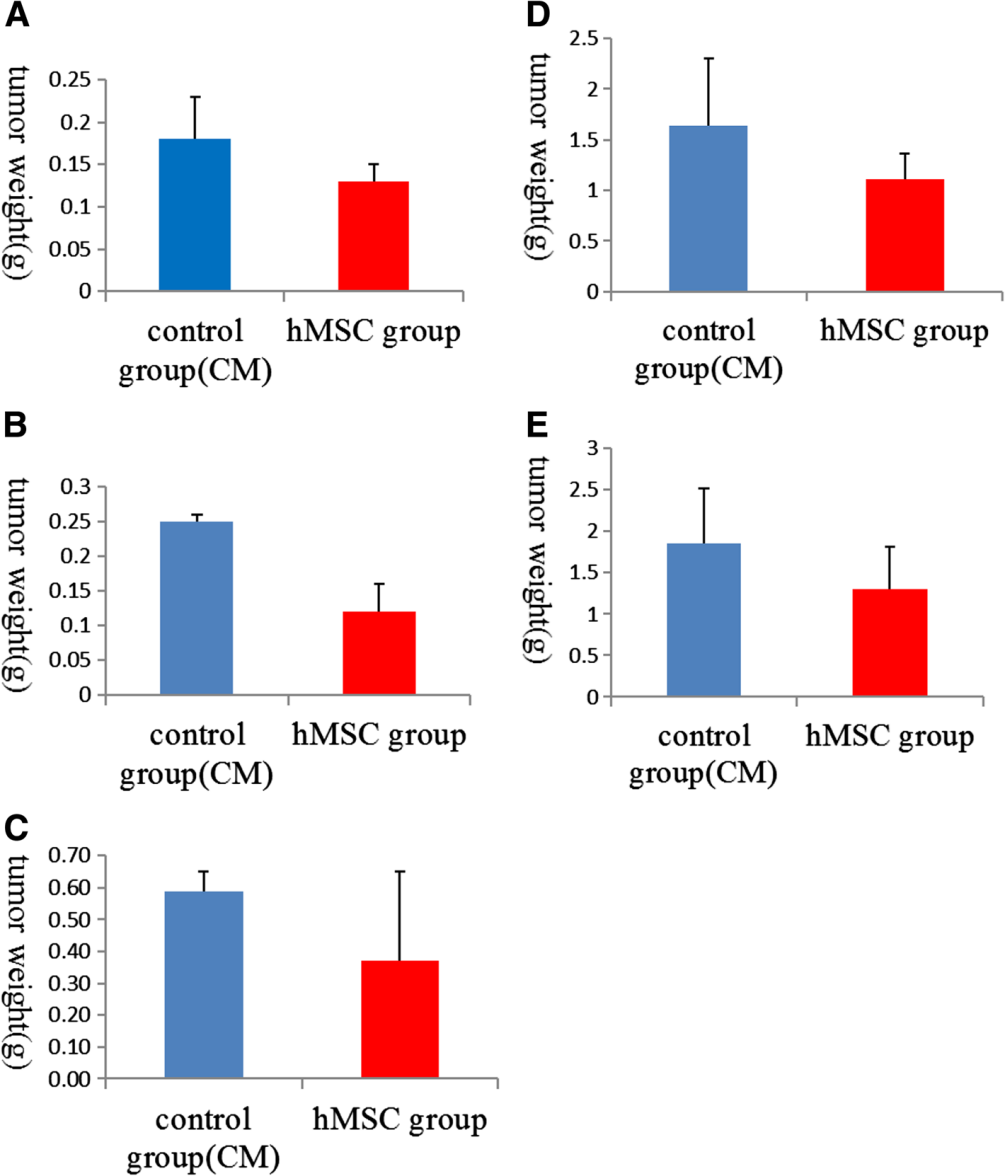
**Tumor tissue *in vitro *weight comparison histogram. (A**, **B)** In the second and third weeks, the tumor weight of human mesenchymal stem cell (hMSC) engraftment groups was significantly lower than that of control groups (*P* <0.01). **(C**, **D**, **E)** In the fourth, fifth and sixth weeks, the difference in tumor weight before and after hMSC intervention was not statistically significant between groups (*P* >0.01).

Tumor tissue *in vitro* weight comparison results were significantly lower in the hMSC engraftment groups than in the control group for the second and third weeks (*P* <0.01), but differences were not statistically significant in the fourth, fifth and sixth weeks between groups (*P* >0.01) (Figure 3).

### RT-PCR determines the expression of OPN, BSP, α-V, Bcl2, Bax and caspase 3 genes in animal models of high-metastatic HCC before and after hMSCs engraftment

**Figure 4:**
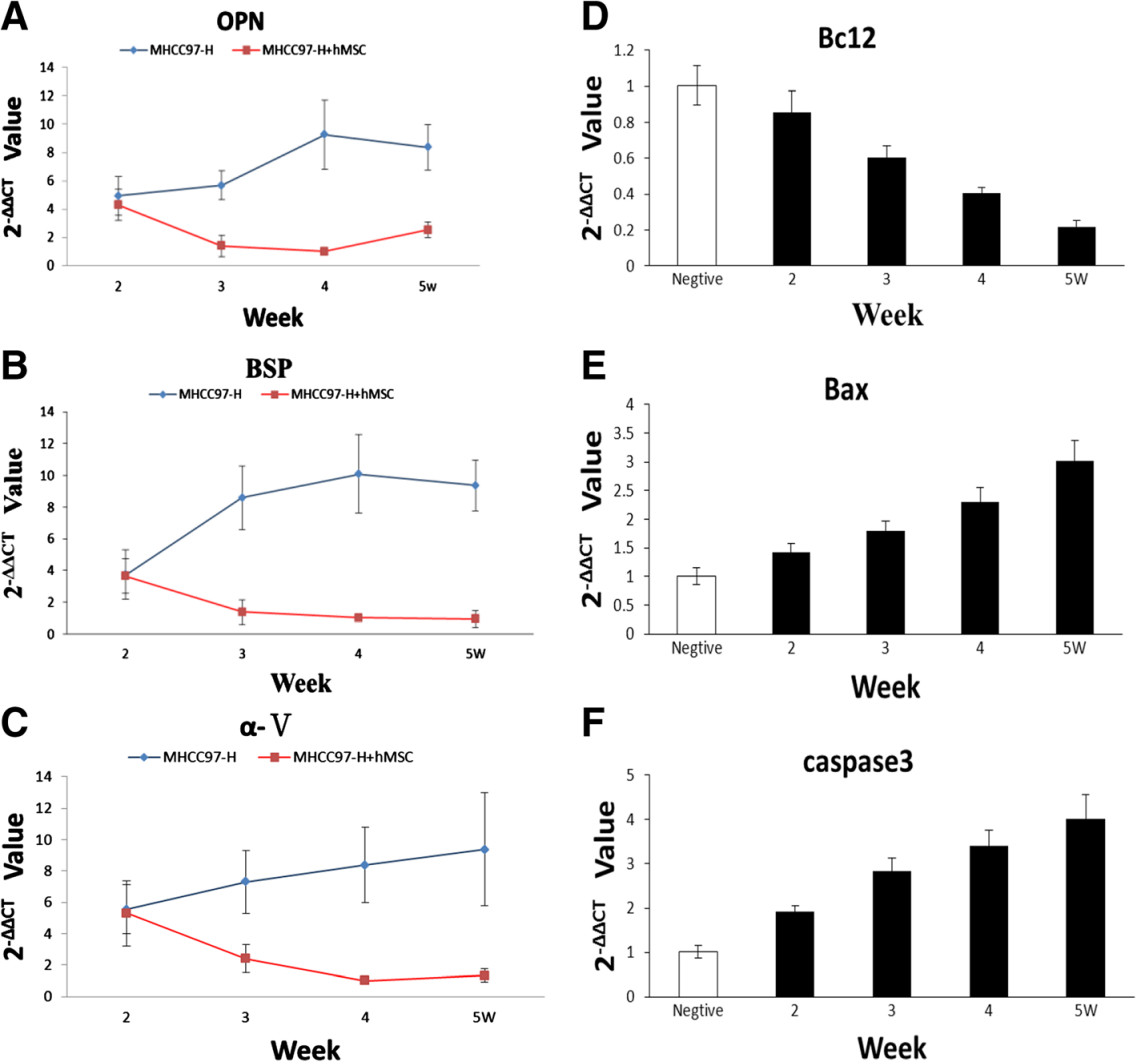
**Gene expression in animal models inoculated with high-metastatic hepatocellular carcinoma cell 97 (MHCC97-H) cells before and after human mesenchymal stem cell (hMSC) engraftment. (A)** The expression of metastasis-related factor osteopontin (OPN) by the tumor tissue specimen over time. **(B)** The expression of metastasis-related factor bone sialoprotein (BSP) by the tumor tissue specimen over time. **(C)** The expression of metastasis-related factor integrin α5 subunit (α-V) by the tumor tissue specimen over time. **(D)** The expression of antiapoptotic gene B cell lymphoma/leukemia-2 (Bcl2) by the tumor tissue specimen over time (note: negative is the control group). **(E**, **F)** The expression of apoptotic gene Bcl-2 associated X protein (Bax) and caspase 3 by the tumor tissue specimen over time (note: negative is the control group).

Following hMSC engraftment, the expression of metastasis-related factors OPN, BSP and α-V gene was downregulated with time. Also, in the fourth week, OPN gene expression in hMSC engraftment groups was significantly lower than in control groups (*P* <0.05). Additionally, in the fourth and fifth week, BSP gene and α-V gene expression in hMSC engraftment groups was significantly lower than in control groups (*P* <0.05). As can be seen from the results, the metastatic potential of tumors was also downregulated after hMSC engraftment, especially in the fourth and fifth weeks (Figure 4A-C).

Following hMSC engraftment, the expression of antiapoptotic gene Bcl2 exhibited an obvious declining tendency, while the expression of apoptotic genes Bax and caspase 3 showed an obvious rising tendency (Figure 4D-F). In the fourth and fifth week, Bcl2 gene expression in hMSC engraftment groups was significantly lower than in negative (control) groups (*P* <0.05). Also in the fourth and fifth week, Bax and caspase 3 gene expression in hMSC engraftment groups was significantly higher than in negative (control) groups (*P* <0.05). As can be seen from these results, the proliferative ability of tumor cells decreased and the apoptotic ability of tumor cells increased after hMSC engraftment.

The results show that the metastatic potential of tumor cells was downregulated after hMSC engraftment, and hMSCs induced more tumor cells apoptosis.

### Correlation analysis between metastasis-related factor expression and apoptotic gene expression in animal models inoculated with MHCC97-H cells after hMSCs engraftment

The expression of metastasis-related factor α-V was significantly positively correlated with the expression of antiapoptotic factor Bcl2 (r = 0.908, *P* <0.01), and negatively correlated with the expression of apoptotic factors Bax (r = −0.803, *P* <0.01) and caspase 3 (r = −0.902, *P* <0.01).

The expression of metastasis-related factor BSP was significantly positively correlated with the expression of antiapoptotic factor Bcl2 (r = 0.890, *P* <0.01), and negatively correlated with the expression of apoptotic factors Bax (r = −0.779, *P* <0.05) and caspase 3 (r = −0.910, *P* <0.01).

**Figure 5:**
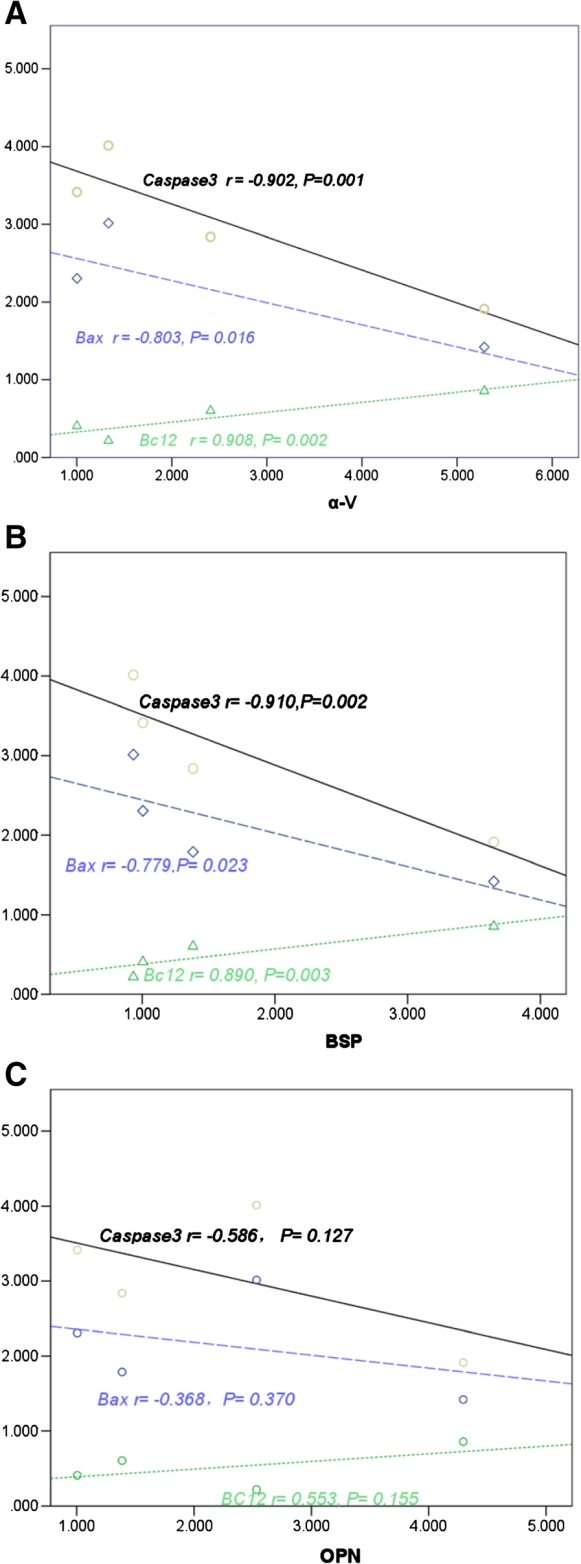
**Correlation analysis (trend charts) between metastasis-related factor expression and apoptotic factor expression in animal models inoculated with high-metastatic hepatocellular carcinoma cell 97 (MHCC97-H) cells after human mesenchymal stem cell (hMSC) engraftment. (A)** Correlation between metastasis-related factor integrin α5 subunit (α-V) and antiapoptotic factor B cell lymphoma/leukemia-2 (Bcl2), and apoptotic factors Bcl-2 associated X protein (Bax) and caspase 3. **(B)** Correlation between metastasis-related factor bone sialoprotein (BSP), and antiapoptotic factor Bcl2 and apoptotic factors Bax and caspase 3. **(C)** Correlation between metastasis-related factor osteopontin (OPN) and antiapoptotic factor Bcl2, and apoptotic factors Bax and caspase 3.

The expression of metastasis-related factor OPN was not significantly correlated with the expression of antiapoptotic factor Bcl2 (r = 0.553, *P* >0.05), and was negatively correlated with the expression of apoptotic factors Bax (r = −0.368, *P* >0.05) and caspase 3 (r = −0.586, *P* >0.05) (Figure 5).

The results suggested that the decrease of proliferation ability of tumor cells can induce the decline of metastatic potential of tumor cells.

### Correlation analysis between apoptotic and metastasis-related gene expression and tumor inhibition rate in animal models inoculated with MHCC97-H cells after hMSC engraftment

The tumor inhibition rate was not significantly correlated with the expression of antiapoptotic factor Bcl2 (r = −0.095, *P* >0.05), and apoptotic factors Bax (r = −0.087, *P* >0.05) and caspase 3 (r = 0.144, *P* >0.05).

**Figure 6:**
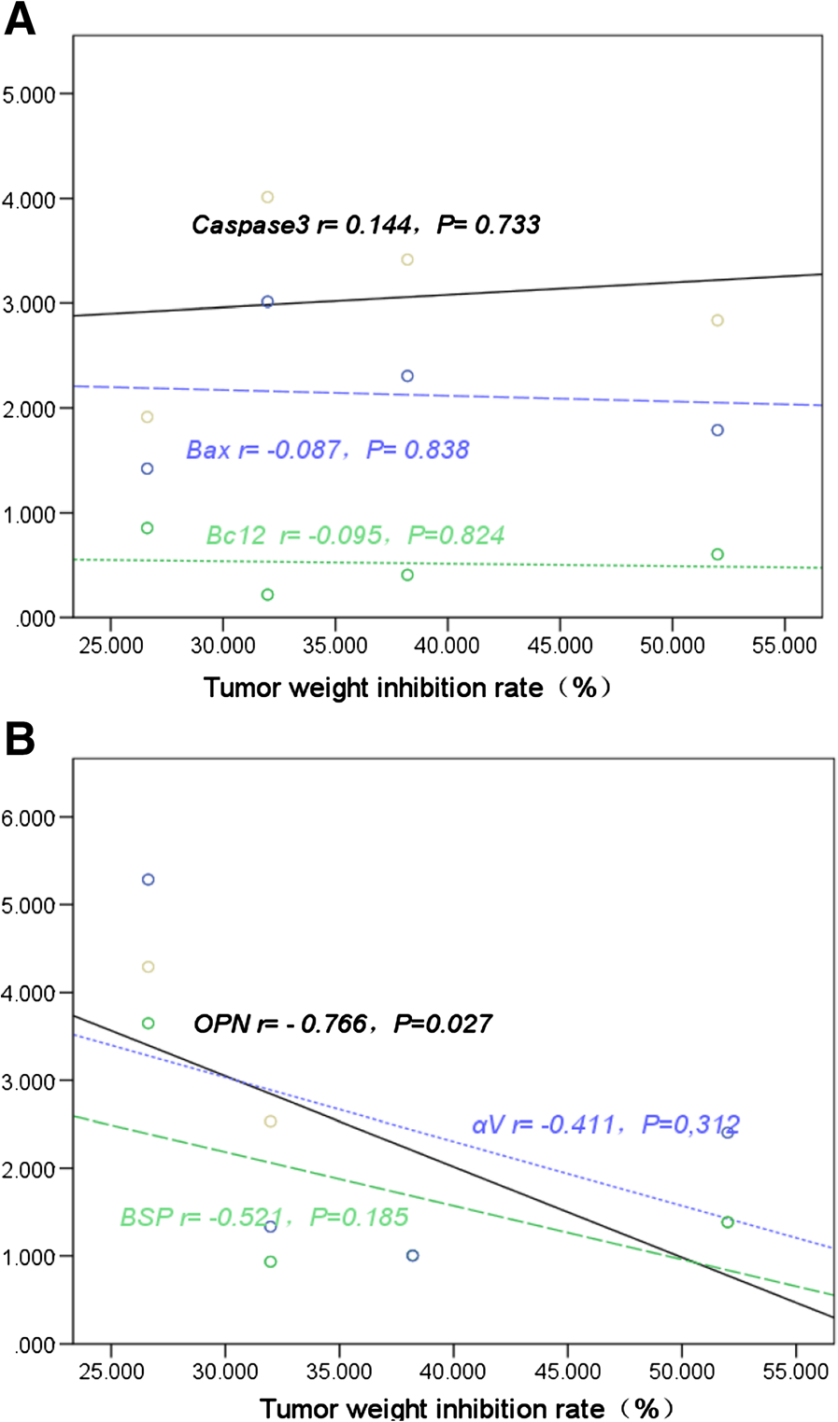
**Correlation analysis (trend charts) between apoptotic and metastasis-related genes expression and tumor inhibition rate in animal models inoculated with high-metastatic hepatocellular carcinoma cell 97 (MHCC97-H) cells after human mesenchymal stem cell (hMSC) engraftment. (A)** Correlation between antiapoptotic factor B cell lymphoma/leukemia-2 (Bcl2), and apoptotic factors Bcl-2 associated X protein (Bax) and caspase 3 gene expression and the tumor inhibition rate in animal models. **(B)** Correlation between metastasis-related gene expression and the tumor inhibition rate in animal models.

The tumor inhibition rate was not significantly correlated with the expression of metastasis-related factors α-V(r = −0.411, *P* >0.05) and BSP (r = −0.521, *P* >0.05) factors, but it exhibited a significant negative correlation with the expression of metastasis-related factor OPN (r = −0.766, *P* <0.05) (Figure 6).

These results suggest that the changes in tumor apoptotic or antiapoptotic genes were not the main factors affecting the tumor inhibition rate. Also, the changes in tumor inhibition rate were not the main factors affecting the metastatic potential of tumor cells.

## Discussion

Liver cancer is one of the most common malignant tumors, and it is the second most common cause of death from cancer in China. Currently, surgical resection remains the most effective approach for treatment of the malignancy. However, the treatment has a high incidence of post-surgical recurrence and metastasis. An estimated 40% to 50% 5-year recurrence rate after surgery is observed in small liver cancers, which is widely recognized to have a good prognosis in clinical practice [6]. Therefore, post-surgical early recurrence and metastasis has become critical as the main hindrance to the long-term survival of patients with liver cancer [6]. It has been found that bone-marrow-derived stem cells are a major extrahepatic source of hepatic stem cells, and bone-marrow-derived stem cells engrafted into the liver tissues of patients can differentiate into liver tissues and stem cells under a specific environment, thereby becoming involving in liver repair and reconstruction [2,3,7]. Autologous bone-marrow-derived stem cells have been engrafted into the injured liver for treatment of decompensated hepatic cirrhosis, which proves that stem cell transplantation is a novel, safe and effective treatment method [8,9]. Stem cell therapy has chemotaxis and homing functions in the treatment of liver cancer, and it presents a high efficacy for promoting the reconstruction of the immune system, removing residual lesions, and preventing recurrence and metastasis [10]. However, there are two completely contradictory views concerning the impact of human bone-marrow-derived mesenchymal stem cells (hMSCs) on hepatocellular carcinomas (HCCs). Some researchers believe that MSCs are a source of circulating progenitors that are able to generate cells of all mesenchymal lineages and cover the cellular demands of injured tissues. MSCs present immunosuppressive and anti-inflammatory features and a high migratory capacity toward inflamed or remodeling tissues. Moreover, experimental evidence has been shown that supports the use of MSCs as a tool in liver fibrosis and hepatocellular carcinoma [4]. However, other researchers hypothesize that MSCs enhance tumor growth but significantly inhibit the invasiveness and metastasis of HCC [11]. Simulation of human tumors in live systems and establishment of tumor models for basic research is a common method in medical research [12]. In mouse models, stem cell transplantation inhibits post-surgical recurrence and metastasis after radical resection of liver cancer, and the antitumor activity is enhanced with the increased chimerism level of hMSC [7].

hMSCs at passages 5 to 8 were used in the present study. Phenotypic identification indicated that the hMSCs at passages 5 to 8 used in this study had all characteristics of hMSCs, which met the requirements for the experiments. Establishment of tumor animal models is a common experimental technique in clinical practice [6]. This study established nude mouse models of HCC with high metastatic potential through inoculation of tumor cells (MHCC97-H cell), and hMSCs were engrafted via the tail vein for intervention treatment of HCC by means of the homing function of hMSCs, so as to observe the therapeutic efficacy of hMSCs against HCC and the effect on high metastatic potential.

The experimental results showed that the hMSCs had an inhibitory effect on HCC in animal models, but the effectiveness of the intervention of hMSCs changed with time. The highest tumor inhibition rate was observed at 3 weeks after hMSC engraftment, and the inhibitory efficiency of hMSCs reduced with time. This demonstrates that hMSCs exhibit antitumor activity in a time-dependent manner.

With regard to the intervention mechanism of hMSCs on liver cancer, studies have reported that hMSCs are found to colonize the liver of mouse models of orthotopic liver transplantation, and can differentiate into hepatocytes that express albumin; in addition, the hMSCs are mainly found in the marginal area of the tumor and rarely present in tumors or normal liver tissues, and a large necrotic area is observed in tumor tissues following hMSC transplantation [12]. In addition to homogeneous gene characteristics and multilineage differentiation potential, hMSCs also have a high efficacy of immunosuppression and anti-infection activity, and the characteristics of migration to inflammatory tissues and remodeling tissues (homing characteristic), which accelerate the repair of injured liver tissues, inhibit immune responses and confer antihepatic fibrosis functions [4]. However, prior to this work, to the best of our knowledge there have been no reports on hMSC antitumor activity in a time-dependent manner.

To further validate the inhibitory effect of hMSCs on HCC, the expression of some apoptosis-associated factors was determined. The Bcl2 family plays a critical role in apoptosis. The Bcl2 family members are classified into two types: antiapoptotic factors, which mainly include Bcl2, Bcl-XL, Bcl-W, Mcl-1 and CED9, and proapoptotic factors, which mainly include Bax, Bak, Bcl-XS, Bad, Bik and Bid. The caspase family plays a very important role in mediating apoptosis, where caspase 3 is a key effector and functions in many apoptosis signaling transduction pathways [13]. From the experimental results, we can draw the conclusion that hMSCs induce greater tumor cells apoptosis, and also, similar to the tumor inhibition rate experiment results, the apoptosis-associated factor test results also show a time-dependent effect, but the timepoints are different. Therefore, the correlation analysis results suggest that the changes in tumor apoptotic or antiapoptotic genes were not the main factors affecting the tumor inhibition rate. Thus, the mechanism of the time-dependent manner of hMSC antitumor activity requires further investigation.

The study determined the expression of metastasis-related factors to observe the effect of hMSCs on metastatic potential of HCC. The results show that the metastatic potential of tumor cells was downregulated after hMSC engraftment. Additionally, the tumor inhibition rate was not significantly correlated with the expression of metastasis-related factors, and the positive correlation with OPN may be due to experimental error. However, the correlation analysis between metastasis-related and apoptosis-associated factor results suggest that the decrease in proliferation ability of tumor cells can induce the decline in metastatic potential of tumor cells. Regardless, the present study also has some shortcomings. The method for determination of tumor metastatic potential in animal models is excessively dependent on molecular biological indexes, while the detection of traditional morphology is neglected.

The present study demonstrates the role of hMSCs in growth and metastasis of HCC tissues through injection of hMSCs via the tail vein, which provides new insight into exploring the relationship between hMSCs and liver cancer metastasis, and may provide a new cytological method for clinical treatment of liver cancer metastasis. It is of great significance to be able to effectively control the pro-proliferative effect and exert the metastatic inhibition ability of hMSCs.

## Conclusions

The highest tumor inhibition rate was observed 3 weeks after hMSC engraftment, and the tumor inhibition rate gradually reduced with time. The metastatic potential of tumor cells was downregulated after hMSC engraftment and hMSCs promoted apoptosis of tumor cells. The decrease in proliferation ability of tumor cells can induce a decline in the metastatic potential of tumor cells. The changes in tumor apoptotic or antiapoptotic genes were not the main factors affecting the tumor inhibition rate. Also, the changes in tumor inhibition rate were not the main factors affecting the metastatic potential of tumor cells.

## Abbreviations

α-V: Integrin α5 subunit; Bax: Bcl-2 associated X protein; Bcl2: B cell lymphoma/leukemia-2; BSP: Bone sialoprotein; HCC: Hepatocellular carcinoma; hMSC: Human bone-marrow-derived mesenchymal stem cell; MHCC97-H: High-metastatic hepatocellular carcinoma cell 97; OPN: Osteopontin; RT-PCR: Quantitative real-time polymerase chain reaction; SPF: Specific-pathogen-free.

## Competing interests

The authors declare that they have no competing interests.

## Authors’ contributions

LR: designed the experiments, acquired and analyzed the data, and drafted the manuscript. SB: designed the experiments. DK: analyzed the data. WM: acquired the data and drafted the manuscript. HL: acquired the data. All authors read and approved the final manuscript.
